# Legal Remedies for Medical Ghostwriting: Imposing Fraud Liability on Guest Authors of Ghostwritten Articles

**DOI:** 10.1371/journal.pmed.1001070

**Published:** 2011-08-02

**Authors:** Simon Stern, Trudo Lemmens

**Affiliations:** 1 Faculty of Law, University of Toronto, Toronto, Ontario, Canada; 2 Faculties of Law and Medicine, University of Toronto, Toronto, Ontario, Canada

**Summary Points**

- Ghostwriting of medical journal articles raises serious ethical and legal concerns, bearing on the integrity of medical research and scientific evidence used in legal disputes.
- Medical journals, academic institutions, and professional disciplinary bodies have thus far failed to enforce effective sanctions.
- The practice of ghostwriting could be deterred more effectively through the imposition of legal liability on the “guest authors” who lend their names to ghostwritten articles.
- We argue that a guest author's claim for credit of an article written by someone else constitutes legal fraud, and may give rise to claims that could be pursued in a class action based on the Racketeer Influenced and Corrupt Organizations Act (RICO).
- The same fraud could support claims of “fraud on the court” against a pharmaceutical company that has used ghostwritten articles in litigation. This claim also appropriately reflects the negative impact of ghostwriting on the legal system.

## Introduction

There are persistent concerns about the influence of the pharmaceutical and device industries on the medical literature, and particularly on the reporting of clinical trials, which can include the distortion of the true evidence base of medical interventions and overestimation of the clinical benefit of a drug used to treat patients [1]. An especially problematic issue involves the industry practice of publishing studies prepared by hired medical writers but signed by academic “guest authors” who are invited to add their names without fulfilling authorship criteria. In this case, “guest authorship” is accompanied by “ghostwriting,” which occurs when a published article fails to acknowledge the original writer or writers' contributions [2]–[4]. Ghostwriting can also occur when an academic research group uses a professional writer to draft an article based on data generated by the group. When the research group retains control of the data and the final analysis, however, there is less of a concern about possible bias in the reporting of the results, and the appropriate remedy in that case is to report explicitly the role and contribution of the medical writer in the article. Here, we concentrate on ghostwriting and guest authorship in industry-controlled research, where several examples have revealed the use of ghostwriters to insert concealed marketing messages favourable to a company's product, and the recruitment of academics as “guest” authors despite not fulfilling authorship criteria [5]–[9].

Commentators have condemned the practice as unethical and unacceptable and have discussed the harms resulting from this form of medical ghostwriting, recommending that journal submissions be policed more aggressively and that the “guest authors” be suitably sanctioned by journals, academic institutions, and regulatory agencies [1]–[14]. However, these recommendations have not yet been widely embraced by the academic institutions, medical journals, and medical licensing organizations that would seem to have the most at stake in curbing this practice. Here, we discuss some of the reasons for this lack of response and suggest that the law may offer a solution, given these other institutions' failure to impose sanctions.

## Concerns about Guest Authorship

Guest authorship is a disturbing violation of academic integrity standards, which form the basis of scientific reliability [15]. The scientific base guiding clinical practice and decision-making is to a large degree formed by the peer-reviewed medical literature. Indeed, pharmaceutical sponsors borrow the names of academic experts precisely because of the value and prestige attached to the presumed integrity and independence of academic researchers. In turn, academics receive considerable credit for publication, thus providing an incentive for their willingness to act as “guests.”

In the legal setting, peer-reviewed articles are credible sources of evidence that may be used in lawsuits to support claims about safety and effectiveness, and hence to determine liability [16]. Industry-controlled publications that are prepared by ghostwriters or that use guest authors may distort perceptions about current knowledge concerning a product's safety and effectiveness. For legal purposes, publication in peer-reviewed journals is one of the criteria that help to make a scientific theory or method admissible as evidence, according to the standards set out by the United States Supreme Court in *Daubert v. Merrell Dow Pharmaceuticals* [17]. By facilitating publication in peer-reviewed journals, guest authorship creates the impression that standards of academic independence and integrity have been satisfied even when they have not, and makes it more likely that the research will be treated as legally admissible even when this is inappropriate.

Publications on which academics appear as guest authors also give credibility to these authors in the legal setting. These articles are sometimes used to establish an expert witness's authority, even when the validity of the research in the article is the very issue under dispute. As a result, the treatment of the guest author as a legal expert may prevent scrutiny of the practice that is being challenged for contributing to serious harm. Numerous studies have shown that industry-sponsored clinical trials are often biased in favor of the sponsor, sometimes in ways that can be detected only with access to the original data and study protocol [8],[9],[18]–[22]. Often, the manipulations that influence the outcome are not visible to the guest author, whose role in the study or article may be minimal and may fall short of authorship criteria that would require involvement in the development and conduct of the study, and final approval of the paper. Thus, guest authors help create the appearance that a study reflects the kind of “scientific methodology” that is required to render evidence admissible under the *Daubert* standard, and in the process they credentialize themselves as expert witnesses who can speak authoritatively about a product's efficacy and safety.

## Curbing Ghostwriting Practices

The International Committee of Medical Journal Editors (ICMJE), in establishing leading standards for biomedical publications, has sought to curb inappropriate and unethical authorship practices by requiring that journals ask detailed questions about what exactly each author has contributed to an article [23]. Editors and editors' associations have a significant interest in preserving the integrity of their journals, and some have detailed sanctions. For example, the World Association of Medical Editors (WAME) says that ghostwriting is “dishonest and unacceptable,” and recommends that on detecting the practice, a journal should “(1) publish a notice that a manuscript has been ghost written, along with the names of the responsible companies and the submitting author; (2) alert the authors' academic institutions, identifying the commercial companies; (3) provide specific names if contacted by the popular media or government organizations; and (4) share their experiences on the WAME Listserve and within other forums” [24]. Similarly, the Committee on Publication Ethics (COPE) recommends that journal editors “adop[t] authorship or contributorship systems that promote good practice (i.e., so that listings accurately reflect who did the work) and discourage misconduct (e.g., ghost and guest authors)” and recommends that when the integrity of research is corrupted, “[e]rrors, [and] inaccurate or misleading statements must be corrected promptly and with due prominence” [25].

Some journals, such as *PLoS Medicine*, have called for bans on future submissions by authors who act as guests, formal retraction if unacknowledged ghostwriting is discovered after publication, and reporting of authors' misconduct to institutions [26]. This may have an impact on academics concerned about their status and future publication options. However, it is unclear whether journals can or even want to monitor the practice adequately. Some editors have stated that their journals are not responsible for policing authorship practices [27]. And because medical journals may gain significant revenue from lucrative advertisement contracts and from selling reprints (including of ghostwritten articles), which industry may use for off-label promotion [28], it is unlikely that medical journals will effectively seek to prevent these practices.

Commentators have also called for academic sanction [12],[29]. But while several established academics have been associated with ghostwritten publications, no public sanctions appear to have been enacted for their behaviour. Various reasons explain an institutional reluctance to take this route: the guest's role in the ghostwritten publications may be unclear; academic institutions may be challenged by their dual commitments to safeguard academic integrity while also protecting their employees against unjust accusation; and universities in particular tend to approach authorship questions with understandable prudence, considering the serious potential impact on academic careers. Academic institutions may also be reluctant to act because ghostwriting cases often involve successful academics who hold positions of power due to their prestige, academic status, publication record, and grant support.

Moreover, institutions may decide not to act because the practices involved in ghost and guest authorship may not be far removed from other common publication practices in academic medicine where laboratory directors, departmental chairs, and supervisors often claim authorship on publications because of those institutional roles rather than by standard authorship criteria [2]. Some clinician-investigators even insist on co-authorship when providing access to patients or samples. Pursuing sanctions for ghostwritten articles may open a Pandora's box, leading to scrutiny of other authorship practices in academia, or to anxiety-laden efforts to justify those practices [30].

Professional organizations, such as State Medical Boards in the US, Colleges of Physicians and Surgeons in Canada, and the General Medical Council in the United Kingdom, could also intervene when evidence of guest authorship by licensed heath care professionals is uncovered, particularly if it involves outright misrepresentation of data [1]. When a physician falsely claims to have analyzed and adequately reported safety and effectiveness data, this can be considered a violation of professional integrity standards and of the commitment to patients and good health care; physicians should know that this may impair clinical care and endanger patients, and they should be sanctioned accordingly [1].

However, these professional organizations have so far failed to issue serious sanctions in the rare cases when an organization has looked into allegations of authorship violations [31]. The reasons for the lack of action may include their general inertia in reacting to new professional challenges and the fact that they may be more preoccupied with other, more traditional violations of professional standards of care, violations of conflicts of interest, and financial fraud. There has also been much criticism of these organizations for their perceived tendency to protect the profession [32],[33]. Finally, for the same reasons as the academic institutions, professional organizations may be uncomfortable about confronting problems of guest authorship and ghostwriting that damage their members.

In light of the lack of institutional responses to curb the practices of ghostwriting and guest authorship and in light of the significance of these practices for the legal system, we suggest that a firm legal response is appropriate.

## Legal Liability for Ghostwriting

An important starting point for a legal response involves the ICMJE uniform guidelines [23] and the authorship forms used by many medical journals based on those guidelines. The theories outlined below apply specifically to journals that require authors to complete and sign such a form as a condition of publication. The guidelines were designed to ensure that authorship credit is reserved to those who have played a significant role in the study's design, conduct, and analysis, and writing of the article. The guidelines set out three criteria, and a person seeking credit as an author must satisfy all three:

1. Substantial contributions to conception and design, acquisition of data, or analysis and interpretation of data;
2. Drafting the article or revising it critically for important intellectual content; and
3. Final approval of the version to be published [23].

Medical journals typically require all authors to confirm in writing that they have satisfied these criteria. “Guest authors” often fail both of the first two requirements, as suggested by evidence that has been revealed in recent class actions involving drugs such as Vioxx (rofecoxib), Prempro (combined estrogen/progestin), and Paxil (paroxetine) [6],[7],[27]. For example, an individual who reads an article and/or offers minor comments has offered nothing substantial under criteria 1 and 2.

The authorship requirements are known not only to named authors but also to readers. The warranty of authorship is an important factor in ascertaining an article's integrity and quality. To see this, we need only ask how readers would react to an article prefaced with a statement that a lead author has refused to sign (or has repudiated) the authorship warranty, and now wishes to clarify the contributions of an industry-based medical writer. Such a statement would significantly undermine the article's credibility [34].

### Guest Authorship as Fraud

The above thought experiment, involving a guest author who admits to playing that role, shows that a false affirmation of authorship is an example of fraud. Fraud occurs when a person makes a knowingly false representation in order to acquire something of value, and harm occurs as a result [35]. In its basic structure, a claim of civil fraud on this basis would take the same form in many countries [36]. However, such a claim is more likely to yield significant damages if numerous plaintiffs can join together to sue in a class action, which may be done more easily in the US than in many other jurisdictions. We therefore draw on US law in this section. Here, the guest's false claim—asserted in the authorship warranty—induces the journal to publish the article, and misleads readers about the scholarly care and scrutiny lavished on the research. The journal gives the guest credit for an article that may serve as a valuable credential, by impressing academic merit committees, grant agencies, conference organizers, and others including judges and juries if the guest later acts as an expert witness [37]. Such recognition may carry reputational and financial value. Arguably, each repetition of the false warranty (implicitly asserted on a CV presented to any of these audiences) is an independent fraud. The journal loses the opportunity to publish an article that would legitimately have satisfied the authorship requirements. The subscribers lose the opportunity to read a legitimate article, and may be led to believe, rely on, and use data from a fraudulent article. If the journal became aware that the lead author was a mere guest, and that the journal's authorship requirements had not been satisfied, the journal would not publish the article.

The characterization of guest authorship as fraud has received limited but important recognition in suits involving the False Claims Act (FCA), which imposes liability on those who cause fraudulent claims to be presented to the US government [38]. For example, in *Strom ex rel. U.S. v. Scios, Inc.*, the US government alleged that the defendants' activities led to the presentation of false Medicare claims [39]. These activities included sponsoring ghostwritten articles purporting to validate off-label use of Natrecor (nesiritide) and, through press releases and the promotional efforts of sales representatives, recklessly encouraging doctors to prescribe the drug for uses that were not medically accepted. Without deciding the merits, the court held that the allegations, if proved, would be sufficient to state a claim under the FCA. In *Strom*, it appears that the unwarranted claims made in the ghostwritten articles, rather than their fraudulent authorship, helped to support the allegations of fraud. This approach has great potential, but it will not always be easy to prove the falsity of ghostwritten research.

As *Strom* shows, the fraud underlying these articles cannot be attributed solely to the guest author, who after all has responded to an invitation. Pharmaceutical companies and medical communications agencies are well aware of the journals' publication requirements. Soliciting and facilitating fraud may amount to conspiracy, and may incur liability on the same grounds as the fraud itself [41]. Such conduct may also constitute fraud under the Racketeer Influenced and Corrupt Organizations Act (RICO) [41]. RICO applies to conspiracies involving at least two prohibited acts within a 10-year period, if those acts “have the same or similar purposes, results, participants, victims, or methods of commission” [41],[42]. The predicate acts for RICO liability include mail and wire fraud, which occur when a fraudulent statement is sent through the mail or by email. If a guest lends her name to two or more articles for the same product, she may satisfy the RICO criteria in several different ways, because the purposes, results, participants, and methods of commission are the same. Civil RICO liability allows plaintiffs to seek treble damages from those violating the statute [41],[42].

Because a journal's readers are all harmed by the fraud, they may sue the guest in a civil RICO class action [43],[44]. One of their harms involves the value of the journal subscription. The subscription price represents the value of a year's worth of articles that conform to the guidelines. Readers would not willingly pay for the fraudulent articles, as shown by the hypothetical example of a guest author who disclaims responsibility for authorship. Whether or not they read the article in question, its publication deprives them of the opportunity to read an article satisfying the journal's requirements, and thus diminishes the value of their subscription. The harm may be measured by reducing the subscription price in proportion to the space devoted to the ghostwritten article. If the subscription costs $100, and the journal publishes 100 articles per year, it could be said that each subscriber suffers a $1 loss from a fraudulent article. The individual loss is small, but the aggregate loss to all subscribers may be significant—particularly if the cost is trebled under RICO.

In addition, some readers access articles on a pay-per-view basis. These readers, too, will assume that the article meets the journal's requirements, and they would also be unlikely to pay if they first saw a disclaimer of authorship responsibility. These purchasers might constitute a distinct subclass in a RICO class action, with damages based on the cost of the download.

To prevail, the plaintiffs would not have to prove individually that they relied on the guest's fraudulent claim. In 2008, the US Supreme Court held that when plaintiffs allege fraud under RICO, they are not required to show that they relied on the defendant's assertions, so long as they were harmed because someone else relied on the fraud (such as the journal editors) [45]. Once a plaintiff establishes that the article was ghostwritten, and shows that he or she paid for a subscription or a download, she has sufficiently established fraud, reliance, and harm for the whole class of RICO plaintiffs.

Why should this approach be directed against guest authors, rather than the others who are complicit in the same fraud? RICO fraud could be added to the claims raised against pharmaceutical companies in negligence suits, but the damages would be low, as against those already available in such cases. But the combination of monetary sanctions and reputational harm might deter academics, and might also deter the medical communications agencies that design these studies and seek impressive names for the byline. Here is a case where the threat of liability—and the uncertainties and distractions that it brings—may be sufficient to discourage those who are not normally sued for harmful drugs, but who help to legitimate the studies that publicize these products.

### Guest-Authored Articles as “Fraud on the Court”

As to the pharmaceutical companies, we propose another approach, also grounded in fraud. Just as the integrity of medical research is a key factor in recognizing false authorship warranties as fraud, the courts' concern about the integrity of their proceedings is key to the doctrine of “fraud on the court.” This doctrine takes a similar form in England, Australia, Canada, India, and many other countries [46]; we focus on US law here because, as explained below, the doctrine had its start in a case that involved a ghostwritten article. A recent formulation of the doctrine defines it as “conduct: 1) on the part of an officer of the court; that 2) is directed to the judicial machinery itself; 3) is intentionally false, willfully blind to the truth, or is in reckless disregard for the truth; 4) is a positive averment or a concealment when one is under a duty to disclose; and 5) deceives the court” [47]. This definition would apply to the use of ghostwritten articles when they are cited by lawyers for those who helped to create the articles or by expert witnesses for those parties. Expert witness testimony comes into court through the agency of lawyers, who are officers of the court. When a pharmaceutical company helps to produce ghostwritten articles and its lawyers cite them in court, the lawyers are, at the very least, reckless about the falsehood and they have a duty to disclose the truth. Remedies for fraud on the court may include a default judgment for the opposing party (when the fraud is revealed during a proceeding), nullification of a judgment or a legal entitlement that was secured with the aid of the fraud, and disbarment of counsel who facilitated the fraud [48].

For a more concrete sense of the doctrine, consider *Hazel-Atlas Glass v. Hartford-Empire Co*. (1944), which seems to be the only ghostwriting case decided by the US Supreme Court [48]. The facts are worth reviewing, because their significance is easily misunderstood—and to the best of our knowledge, the case has not been cited by any commentators on medical ghostwriting. In 1926, Hartford tried to patent a method of molding glass. Faced with skepticism from the Patent Office, Hartford's employees wrote an article lauding their method as an important advance, and then found an author for it in William Clarke, president of the Flint Glass Workers' Union. After publishing the article in a trade journal, Hartford cited it in their patent application, and the patent was granted. In 1928, Hartford sued Hazel, a competing glass manufacturer, for infringing the patent, but lost at trial. On appeal, Hartford leaned heavily on the spurious article. Hazel doubted its legitimacy, and interviewed Clarke, but he refused to acknowledge the truth. The court of appeals ruled for Hartford, quoting from the article as evidence of the patent's novelty and utility. The truth came to light 9 years later, when Hartford disclosed its files during an antitrust action. In 1944, the Supreme Court vacated the prior judgment, sanctioning Hartford's use of the article as a fraud on the court. The Court also nullified Hartford's patent, and the Hartford lawyers who had used the spurious article were disbarred from practice before the Patent Office [49].

In explaining why Hartford's actions merited sanction, the Supreme Court offered several observations that apply with equal force to current examples of medical ghostwriting. The Court stated that using spurious claims of authorship to legitimate claims before the Patent Office and the courts “is a wrong against the institutions set up to protect and safeguard the public” [48]. Precisely the same could be said about ghostwritten articles published in medical journals through false warranties of authorship. The courts are among the institutions wronged by such practices, which may lead judges to treat the ghostwritten publications as evidence that is legally admissible according to the *Daubert* requirements, as noted above [17]. Hartford argued that it was impossible to prove that the article was responsible for their legal victory, but the Court rejected that argument: “Hartford's officials and lawyers thought the article material. They . . . went to considerable trouble and expense to get it published. . . . [T]hey urged the article upon the Circuit Court and prevailed. They are in no position now to dispute its effectiveness” [48]. We might expect pharmaceutical defendants to minimize the evidentiary role of ghostwritten articles today, and the same answer would be appropriate.

Ghostwritten articles are not created and developed primarily for legal purposes; rather, they are used to publicize and market drugs. However, a restriction on the legal use of articles to which guest authors have added their name could significantly diminish their overall value. They are often used in litigation to support the manufacturer's arguments about a drug's efficacy and safety, or to establish a record of scientific acceptance for *Daubert* purposes, or to credentialize an expert witness. Each of those uses, if attempted by a party that had helped to create the article, could risk sanction. The articles could still be used to promote drugs, but if litigation should arise, the defendant's arsenal of responses would be limited.

## Conclusion

The false respectability afforded to claims of safety and effectiveness through the use of academic investigators risks undermining the integrity of biomedical research and patient care. This integrity also underpins the use of scientific evidence in the courtroom. Whether publications with academic guest authors are factually accurate is irrelevant. In *Hazel-Atlas*, Hartford insisted that the article's claims were true, attribution issues notwithstanding. The Supreme Court found this argument unavailing: “Truth needs no disguise. The article, even if true, should have stood or fallen under the only title it could honestly have been given—that of a brief in behalf of Hartford, prepared by Hartford's agents, attorneys, and collaborators” [48]. Today, as in 1944, one might expect the sponsors of ghostwritten articles to treat the question of false authorship as an insignificant detail that merits no legal sanction. The US Supreme Court's comments provide a sufficient rebuttal to such claims.

## Abbreviations

RICO: Racketeer Influenced and Corrupt Organizations Act

## References

[1] Lemmens T (2004). Leopards in the temple: restoring scientific integrity to the commercialized research scene.. JL Med Ethics.

[2] Gøtzsche PC, Kassirer JP, Woolley KL, Wager E, Jacobs A (2009). What should be done to tackle ghostwriting in the medical literature?. PLoS Med.

[3] Sismondo S (2009). Ghosts in the machine: publication planning in the medical sciences.. Soc Stud Sci.

[4] Matheson A (2008). Corporate science and the husbandry of scientific and medical knowledge by the pharmaceutical industry.. BioSocieties.

[5] Healy D, Cattell D (2003). Interface between authorship, industry and science in the domain of therapeutics.. Br J Psychiatry.

[6] Ross JS, Hill KP, Egilman DS, Krumholz HM (2008). Guest authorship and ghostwriting in publications related to rofecoxib: a case study of industry documents from rofecoxib litigation.. JAMA.

[7] Fugh-Berman AJ (2010). The haunting of medical journals: how ghostwriting sold ‘HRT.’. PLoS Med.

[8] Jureidini JN, McHenry LB, Mansfield PR (2008). Clinical trials and drug promotion: Selective reporting of Study 329.. The International Journal of Risk and Safety in Medicine.

[9] Johnson KR, Lassere MND (2008). Guest authorship, mortality reporting, and integrity in rofecoxib studies.. JAMA 300: 900; author reply.

[10] Flanagin A, Carey LA, Fontanarosa PB, Phillips SG, Pace BP (1998). Prevalence of articles with honorary authors and ghost authors in peer-reviewed medical journals.. JAMA.

[11] Smith R (2005). Medical journals are an extension of the marketing arm of pharmaceutical companies.. PLoS Med.

[12] Moffatt B, Elliott C (2007). Ghost marketing: pharmaceutical companies and ghostwritten journal articles.. Perspect Biol Med.

[13] Gøtzsche PC, Hróbjartsson A, Johansen HK, Haahr MT, Altman DG (2007). Ghost authorship in industry-initiated randomised trials.. PLoS Med.

[14] United States Senate Committee on Finance (2010). Ghostwriting in medical literature minority staff report, 111th Congress, Sen..

[15] Biagioli M (2000). Rights or rewards? changing contexts and definitions of scientific authorship.. Journal of College and University Law.

[16] Edmond G (2008). Judging the scientific and medical literature: some legal implications of changes to biomedical research and publication.. Oxford Journal of Legal Studies.

[17] Daubert v. Merrell Dow Pharmaceuticals. 509 U.S. 579 (1993).

[18] Lexchin J (2003). Pharmaceutical industry sponsorship and research outcome and quality: systematic review.. BMJ.

[19] Bekelman JE, Li Y, Gross CP (2003). Scope and impact of financial conflicts of interest in biomedical research: a systematic review.. JAMA.

[20] Schott G, Pachl H, Limbach U, Gundert-Remy U, Ludwig W-D, et al. (2010). The financing of drug trials by pharmaceutical companies and its consequences. Part 1: a qualitative, systematic review of the literature on possible influences on the findings, protocols, and quality of drug trials.. Dtsch Arztebl Int.

[21] Schott G, Pachl H, Limbach U, Gundert-Remy U, Lieb K (2010). The financing of drug trials by pharmaceutical companies and its consequences: part 2: a qualitative, systematic review of the literature on possible influences on authorship, access to trial data, and trial registration and publication.. Dtsch Arztebl Int.

[22] Caplovitz A (2006). Turning medicine into snake oil, how pharmaceutical marketers put patients at risk..

[23] International Committee of Medical Journal (2009). Uniform requirements for manuscripts submitted to biomedical journals: ethical considerations in the conduct and reporting of research: authorship and contributorship.. http://www.icmje.org/ethical_1author.html.

[24] World Association of Medical (2005). Ghost writing initiated by commercial companies.. http://www.wame.org/resources/policies#ghost.

[25] Committee on Publication Ethics (COPE) (2011). COPE code of conduct.. http://www.publicationethics.org/files/Code_of_conduct_for_journal_editors_Mar11.pdf.

[26] The *PLoS Medicine* (2009). Ghostwriting: the dirty little secret of medical publishing that just got bigger.. PLoS Med.

[27] McHenry LB, Jureidini J (2008). Industry-sponsored ghostwriting in clinical trial reporting: a case study.. Accountability in Research.

[28] Smith R (2006). Lapses at the New England journal of medicine.. J R Soc Med.

[29] Sismondo S (2007). Ghost management: how much of the medical literature is shaped behind the scenes by the pharmaceutical industry?. PLoS Med.

[30] Moffat B (2011). Responsible authorship: why researchers must forgo honorary authorship.. Account Res.

[31] Baty P (10 November 2009). Academic made ‘untrue’ declaration about ‘full access’ to research material, GMC finds.. http://www.timeshighereducation.co.uk/story.asp?storycode=409013.

[32] Irvine D (2006). A short history of the General Medical Council.. Medical Education.

[33] Picard A (19 August 2007). Does self-regulation work for the medical profession?. The Globe and Mail.

[34] Lacasse JR, Leo J (2011). Knowledge of ghostwriting and financial conflicts-of-interest reduces the perceived credibility of biomedical research.. BMC Research Notes.

[35] Rakes v Life Investors Ins..

[36] R. v. Théroux, [1993] 2 S.C.R. 5 [Canada]; Magill v. Magill, (2006). 231 A.L.R. 277 (HC) [Australia]; Barlow Clowes International Ltd v Eurotrust International Ltd (2005) [United Kingdom]; Commissioner of Customs (Preventive) v..

[37] Edmond G (2007). Supersizing Daubert science for litigation and its implications for legal practice and scientific research.. Villanova Law Review.

[38] 31 U.S.C. § 3729

[39] Strom ex rel. U.S. v. Scios, Inc., 676 F. Supp.2d 884 (N.D. Cal. 2009)

[40] 18 U.S.C. § 2

[41] 18 U.S.C. §§ 1961-62

[42] Sedima, S.P.R.L. v. Imrex Co., 473 U.S. 479 (1985)

[43] Am. Nat'l Bank & Trust Co. v. Haroco, Inc., 473 U.S. 606 (1985)

[44] Holmes v. Sec. Investor Prot. Corp., 503 U.S. 258 (1992)

[45] Bridge v. Phoenix Bond & Indemnity Co., 128 S. Ct. 2131 (2008)

[46] (1993). 84 C.C.C. (3d) 422 (B.C.C.A.) [Canada]; S.P. Chengalvaraya Naidu v..

[47] Johnson v

[48] Hazel-Atlas Glass Co. v. Hartford-Empire Co., 322 U.S. 238 (1944)

[49] Hatch v. Ooms, 69 F. Supp. 788 (D.D.C.1947), rev'd sub nom. Dorsey v. Kingsland, 173 F.2d 405 (D.C. Cir. 1949), rev'd, 338 U.S. 318 (1949)

